# Striking Differences in Kendo Headgear

**DOI:** 10.7759/cureus.61723

**Published:** 2024-06-05

**Authors:** An Phuc D Ta, Megan D Hsu, Harrison Chu, Audrey San Pedro, Hillary Chu, Alexis Leo, Satori Iwamoto, Hao Chen, Gary Chu

**Affiliations:** 1 College of Medicine, California Northstate University, Elk Grove, USA; 2 Respiratory Medicine, Yokohama City University Hospital, Yokohama, JPN

**Keywords:** safety standards, helmet design, concussion prevention, protective gear, helmets, kendo

## Abstract

Background: Kendo, a martial art developed by the samurai, is rooted deep in Japanese culture with traditional armor that has seen little change over the past centuries. Despite its century-old design, kendo helmets are manufactured without third-party testing to verify their quality and effectiveness against head trauma.

Objective: To evaluate the effectiveness of different helmet stitching patterns and padding materials in mitigating impact forces that could lead to sports-related concussions (SRC) in kendo, and to assess variations in safety performance across different genders and kendo ranks (Dan and Kyu).

Methods: We collected data from 10 kendo practitioners (six males and four females), analyzing over 4,000 strikes using shinai on a sensor-equipped mannequin. Various helmet stitching patterns (ranging from 2 mm to 9 mm) and padding types (polyurethane-based and different thicknesses of cotton-based pads) were tested under controlled conditions simulating realistic impacts encountered in kendo practice.

Results: The results indicated that helmets with wider stitching patterns (e.g. 8 mm and 9 mm) generally offered better energy absorption, exhibiting statistically significant lower mean g-forces with a 95% confidence interval compared to tighter patterns (2 mm, 4 mm, 6 mm, and 8 mm x 2 mm) (p < 0.001). Additionally, the polyurethane-based padding outperformed cotton-based padding by a statistically significant reduction of impact force (p < 0.001). Significant differences in striking force were also observed between genders and ranks, with male and higher-rank (Dan) practitioners delivering stronger impacts (both p < 0.001).

Conclusions: This study highlights the critical influence of helmet stitching patterns and padding materials on the protective capabilities against concussions in kendo. Even though helmets with narrower stitching patterns cost more, helmets with wider stitching patterns and polyurethane padding material provide enhanced safety benefits. We do not know how the difference in striking force between genders and ranks affects the outcome of a kendo match.

## Introduction

Kendo, a martial art developed by the Japanese samurai centuries ago, is rooted deep in Japanese culture and tradition. Over the past 100 years, little has changed in its armor design. While independent organizations exist to ensure the quality of the shinai, a bamboo sword, that is used in kendo, no third-party ever existed to verify or test the effectiveness of kendo armor manufactured, even in modern-day Japan [1-3]. In contemporary day sports, concussions remain a significant concern in contact sports, occurring with or without protective headgear [4]. Although helmets can offer ample protection against traumatic brain injuries (TBIs), different types of impacts can still cause various head movements, such as rotational forces, which can lead to other forms of head injuries [5]. Recent studies have found that competitive soccer has been the leading cause of concussions among female high school students, following closely behind high school football [6]. Compared to other contact sports and martial arts, kendo is considered a relatively safe sport with a lower overall injury rate and most injuries consisting of contusions, abrasions, or sprains [7]. However, sports-related concussions (SRC) are still reported in kendo. In Fukuoka University’s 2020 annual health report, at least seven students from the university’s kendo club sustained concussion injuries during that school year [8]. With this in mind, it is pertinent to consider the effectiveness of helmets in preventing and protecting against SRCs [9]. Helmets are designed in a way where impact force can be distributed over a larger area. The supporting foam linings within the helmets further reduce the impact by extending the time that it takes for the head to slow down, thereby making the movement less sudden [5]. Given the scarcity of research on kendo concussion prevention, our team seeks to determine the optimal padding material and stitch patterns that best reduce concussion risk. We attempt to evaluate the effectiveness of different helmet stitching patterns and padding materials in mitigating impact forces that could lead to SRCs in kendo and to assess variations in safety performance across different genders and kendo ranks (Dan and Kyu). Do pricier kendo helmets offer more head protection than an entry-level helmet? Are modern paddings better than traditional cotton pad inserts?

Back in feudal Japan, martial arts like kendo were primarily practiced by samurai for survival and security, but they underwent a significant transformation during the Edo period (1603-1867) into pursuits focused on mental and self-improvement [10]. Following World War II, the Japanese government increased efforts to promote Japanese culture globally, including kendo, as part of cultural diplomacy, resulting in its international spread spearheaded by Japanese immigrants establishing kendo dojos (training halls) worldwide [10,11]. Technological advancements and improved transportation have further facilitated international kendo exchanges such as seminars and tournaments, enabling kenshi (swordsmen) from different countries to participate and learn from each other [11]. The word kendo originates from ken (sword) and do (way or path), reinforcing the philosophy of the “way of the sword” [12]. Compared to other full-contact sports in Europe or America, kendo emphasizes scoring points through targeted strikes using the shinai, with matches requiring two points for a victory [13]. The validity of the strikes is evaluated by multiple shinpan (referee/judge) on various factors including fighting spirit, posture, and zanshin (continued alertness) [14-16].

Today, approximately six million people practice kendo worldwide, with around a third of practitioners based in Japan [13]. Despite regional variations, internationalization efforts of budo (martial arts) prioritize maintaining the essential nature of kendo, including its ranking system of Dan and Kyu levels. Kyu levels denote junior rank, comparably equivalent to “color belts” karate, ranking, ranging from 10th kyu (lowest) to 1st kyu (highest). Conversely, dan levels signify senior ranks, with 1st dan considered equivalent to a first-degree black belt and progressing up to 10th dan [17]. As kendo gains more popularity, modifications to kendo protective gear have been aimed at reducing equipment weight to increase agility. In response to safety concerns, the All-Japan Kendo Federation has stepped in to set standards for the tip thickness of shinai (bamboo sword) and the length of kendo robe sleeves [4]. When selecting kendo men (helmets), there are several styles, qualities, and brands available [18]. Between hand-stitched and machine-stitched helmets, hand-stitched products are pricier as they can take up to several years to craft. Both stitching styles follow the same system that is defined by the distance between each stitch (e.g., 2 mm, 4 mm, 6 mm, 8 mm, and 9 mm). These helmets are available in various stitch densities, which inversely affect cost and protection. Denser stitches offer more resilience and shape retention but may reduce impact absorption and increase energy transfer to the user’s head [18-19]. Ultimately, the choice of helmet will depend on the kendo practitioner’s personal preferences, financial capabilities, and safety considerations. As the level of interest and participation in kendo grows, it becomes imperative to ensure that kendo protective equipment undergoes thorough testing to reduce the number of sports-related injuries.

## Materials and methods

**Figure 1:**
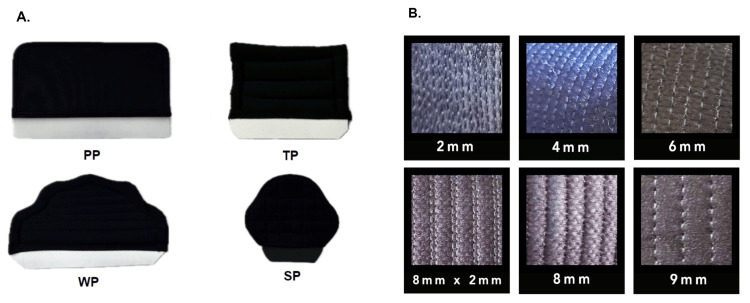
Padding insert materials (A) Four types of protective padding inserts were placed inside the helmet. Polyurethane-based pad (PP), thick pad (TP), cotton-based pad, wide pad (WP), cotton-based, small pad (SP), cotton-based. (B) Six stitching lengths of helmets from most dense to least: 2 mm, 4 mm, 6 mm, 8 mm x 2 mm hybrid, 8 mm, and 9 mm.

This study comprised 10 kendo practitioners, including six males and four females, of dan or Kyu-level ranking contributing to a dataset of over 4,000 proper men strikes (straight downwards). All participants were recruited following Institutional Review Board (IRB)-approved protocols (between 18 and 60 of age, not currently pregnant, in good health and physical shape). For this experiment, commercial off-the-shelf helmets and helmet padding inserts were utilized. The padding insert materials studied included polyurethane-based and cotton-based, thick cotton-based, wide cotton-based, and small cotton-based (Figure 1). Furthermore, various helmet stitching patterns were examined, including 2 mm, 4 mm, 6 mm, 8 mm x 2 mm hybrid, 8 mm, and 9 mm stitch patterns.

**Figure 2:**
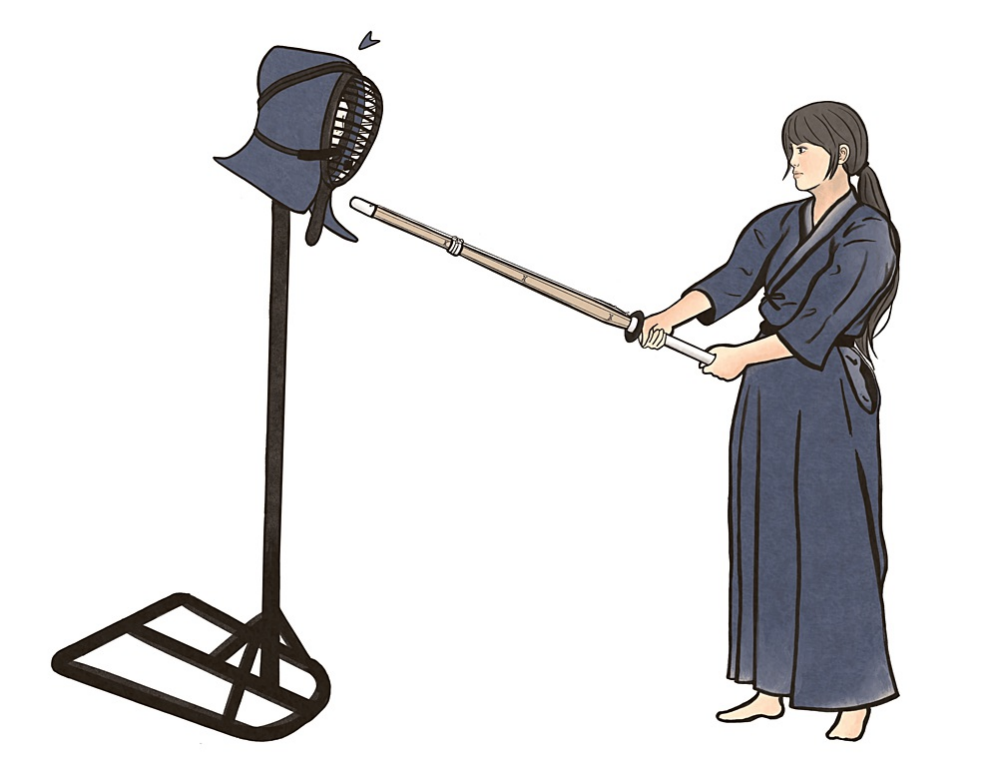
Study setup Kendo practitioner preparing to strike the mannequin, equipped with protective gear and embedded sensor. The arrow indicates the striking target location. The figure was drawn by Megan Hsu.

In a blinded setup, the kendo practitioners used shinai to give proper men (straight down) strikes on a mannequin head. The mannequin head, representing the average male head size of height 33 cm and circumference 57 cm [20], was mounted on a metal post at a height of 175 cm. A sensor measuring linear acceleration (g-force) was placed within the mannequin head and surrounded by silicone material to simulate the natural position of the brain (Figure 2). In combination with the sensor, the NET PLAYZ Combat Force Tracker application (NET PLAYZ, USA) computed and displayed measured linear acceleration on a monitor.

The experimental arrangements included the mannequin head equipped with: No protective helmet; six helmets of the same size with stitching patterns from 2 to 9 mm and no padding inserts; and a 4 mm helmet with one of four possible helmet padding inserts: polyurethane-based pad (PP), thick pad (TP), wide pad (WP), or small pad (SP).

In this research study, 62.4 and 102.5 g-forces were designated as the cutoff point, representing the lowest reported linear accelerations indicating concussion risk for young athletes and adults, respectively [21]. We analyzed the differences in concussion protection across four padding types and six helmet designs using one-way ANOVA tests, while unpaired, two-tailed t-tests compared striking forces among Dan and Kyu-level practitioners, as well as between male and female participants. All statistical tests and subsequent graphical analyses were performed using GraphPad Prism 10.

## Results

**Figure 3:**
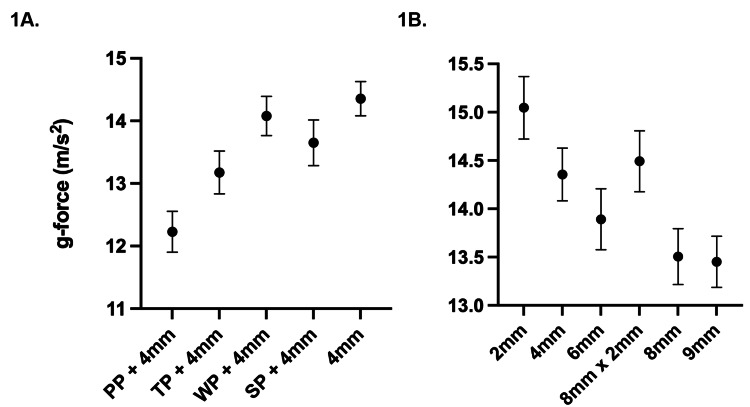
(A) Various kendo paddings with 4 mm helmets are effective at reducing concussions with varying efficacies. Linear acceleration received by the pads (p < 0.001). (B) Various kendo helmets are effective at reducing concussions. Linear acceleration received by the pads (p < 0.001). PP polyurethane = PP 8 mm x 2 mm is a hybrid helmet. Average linear acceleration is represented by center dots with 95% confidence intervals. PP: polyurethane-based pad; TP: thick cotton-based pad; WP: wide cotton-based pad; SP: small cotton-based pad.

The PP with a 4 mm helmet received the lowest linear acceleration with a 95% CI of (11.9-12.6), followed by the TP (12.8-13.5), small (SP) (13.8-14.4), and cotton-based WP (13.3-14.0). When the 4 mm helmet was combined with the PP or TP, an unpaired two-tailed t-test indicated significant improvement in reducing linear acceleration versus a 4 mm helmet alone (p < 0.001) (Figure 3A).

The 9 mm helmet received the lowest linear acceleration (13.2-13.7) followed by the 8 mm helmet (13.2-13.8), 6 mm helmet (13.6-14.2), 4 mm helmet (14.1-14.6), 8 mm x 2 mm hybrid (14.2-14.8), and then 2 mm helmet (14.7-15.4) (Figure 3B). Using ANOVA tests, the differences between g-forces received by the various paddings and helmets were statistically significant (p < 0.001) (Table 1). 

**Table 1:** Descriptive statistics of linear acceleration for different paddings, helmets, skill levels, and gender. ANOVA test was utilized to compare g-forces received by the pads and helmet. All tested paddings were under the 4 mm helmet. An unpaired, two-tailed t-test was utilized to compare g-forces from Dan and Kyu-ranked practitioners, and male versus female practitioners. Polyurethane: polyurethane-based pad; n: sample size, mean linear accelerations are in g-forces (m/s^2^).

	N	Mean (SD)	95% CI
Padding	p < 0.0001		
Polyurethane	361	12.2 (3.16)	(11.9-12.6)
Thick	363	13.2(3.31)	(12.8-13.5)
Wide	375	14.1 (3.09)	(13.8-14.4)
Small	357	13.7 (3.50)	(13.3-14.0)
Bogu	p < 0.0001		
2 mm	410	15.0 (3.34)	(14.7-15.4)
4 mm	426	14.4 (2.87)	(14.1-14.6)
6 mm	415	13.9 (3.27)	(13.6-14.2)
8 mmx 2 mm	415	14.5 (3.27)	(14.2-14.8)
8 mm	416	13.5 (3.00)	(13.2-13.8)
9 mm	422	13.5 (2.77)	(13.2-13.7)
Skill-level	p = 0.0003		
Dan	619	13.7 (3.53)	(13.4-13.9)
Kyu	837	13.0 (3.16)	(12.8-13.2)
Gender	p < 0.0001		
Male	2807	14.2 (3.23)	(14.0-14.3)
Female	1458	13.4 (3.15)	(13.2-13.6)

**Figure 4:**
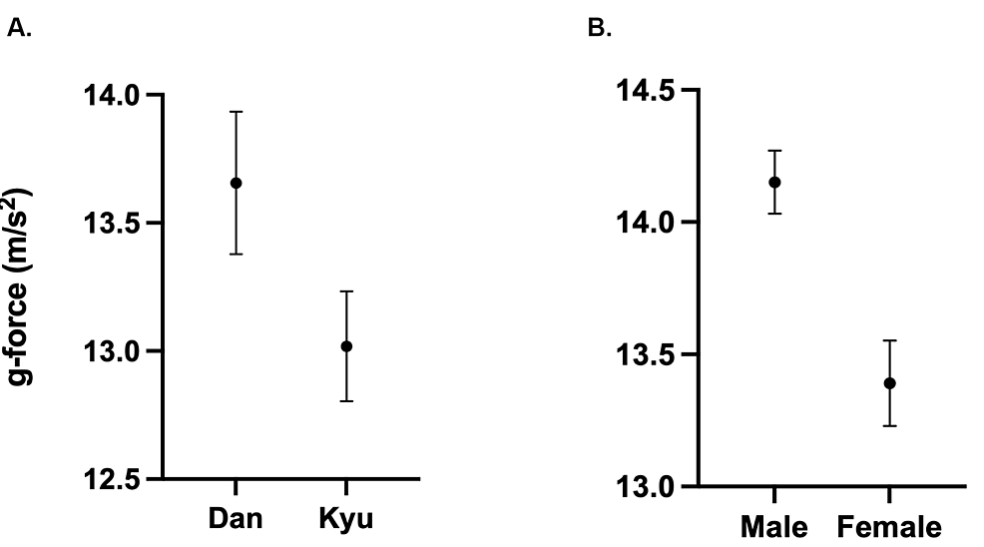
(A) Linear accelerations from Dan practitioners are higher than from kyu practitioners (p < 0.001). (B) Linear accelerations from male kendo practitioners are greater than female practitioners (p < 0.001). The average is represented by center dots with 95% confidence intervals.

Dan-level practitioners (two males, one female) hit with greater force on average than Kyu-level practitioners (four males, three females) with (13.4-13.9) g-forces compared to (12.8-13.2) g-forces of kyu practitioners (Figure 4A). Male practitioners hit on average (14.0-14.3) harder than female practitioners (13.2-13.6) (Figure 4B). Unpaired, two-tailed t-tests of bogu linear acceleration suggest significant differences in striking force between Dan and Kyu-level practitioners (p < 0.001) and male and female practitioners (p < 0.001).

Linear accelerations of padless helmets with 2-9 mm stitching patterns and all paddings with 4 mm helmets were well below the threshold risk of concussion at 62.4/102.5 g-forces (youth athletes/adults) (Figure 3). They performed significantly better than having no helmet, which measured at 23.6 ± 3.25 g-forces (p < 0.001). The PP + 4 mm helmet combination provided the most protection out of the helmets and paddings used in the study (Figure 3).

## Discussion

The findings of this study confirm that commercially available helmets of stitching patterns 2-9 mm sufficiently protect against concussions against proper helmet hits (p < 0.001). When comparing striking forces received by the various helmets, we discovered a trend of wider stitching patterns offering greater protection (Figure 3B). Specifically, the 8 mm and 9 mm helmet stitching patterns provided the greatest protection among the helmets (p < 0.001) (Figure 3B). This observation may be attributed to the enhanced impact absorption potential of wider stitch patterns due to the increased distance between stitches, which allows for more padding material to absorb impact. However, this comes at the expense of reduced helmet shape retention, which affects the helmet fit [18,19]. Conversely, tighter stitching patterns may result in less padding impact absorption. This is consistent with the 2 mm helmet offering the least comparative protection among the other tested stitching patterns (Figure 3B). Nonetheless, it is important to reiterate that regardless of the stitch pattern variant, all tested helmets demonstrated sufficient protection in reducing the risk of concussions from straight downward strikes to the head.

Force impacts on the exterior of a helmet from shinai strikes will naturally dissipate energy inside the helmet, to the padding, the skull, and then the brain [22]. When comparing the paddings, the PP provided on average the most protection, followed by the TP, SP, and WP (Figure 3A), suggesting that PP dissipates more energy before its residual reaches the brain. Moreover, when the 4 mm helmet was fitted with the PP or TP, the overall protection was even greater compared to the 4 mm helmet alone (p < 0.001). The material of PP, polyurethane, has been commercially used as paddings in other sports and combat headgear. In comparison, the SP + 4 mm helmet and WP + 4 mm helmet are within the same 95% CI as the 4 mm helmet alone, suggesting that the SP and WP may not significantly enhance protection. Whereas, the PP and TP significantly improved protection when combined with the 4 mm helmet, with the PP providing the highest increase in protection, i.e.,17.3%. These findings suggest that both the polyurethane pad and the thick cotton-based pad are strong considerations when purchasing protective gear. These findings have not been reported in the literature before and offer valuable insights for consumers looking to purchase kendo equipment.

One additional factor to consider is the size of the pads since extra bulky pads can embed space otherwise occupied by air. Mathon et al. mentioned that air protects the head due to its capacity for deformation, instantaneous absorption, and the distribution of forces throughout the compressed volume [23]. The energy redistributes over a longer period resulting in lower maximal acceleration [22]. When struck with shinai, more bulky helmet pads have increased maximal linear acceleration compared to a less bulky identical pad due to the differences in air space. The fit of a helmet plays a crucial factor in the amount of force transferred to the brain. Pads that disrupt the overall fit of the helmet may result in more concussions whereas well-fitted pads have improved protection [24].

We found striking differences between Dan and Kyu-level practitioners, and gender (Figure 4). On average, Dan-level practitioners delivered strikes with significantly greater force than Kyu-level practitioners (p < 0.001) (Figure 4A), with an 8.8% max increase. Based on the CI spread, male practitioners strike harder than female practitioners by about 7% (p < 0.001) (Figure 4B). This trend is consistent with findings from prior studies in other sports where men historically perform better than women [25]. While the 7% difference may seem small, it can represent the spread between first place and 35th place, such as in the 2022 Winter Olympics Men’s downhill ski race [26]. Nonetheless, for kendo, it remains uncertain whether this difference in striking force could affect the outcome of a kendo match between a male and a female practitioner.

The biggest limitation of our study was the inconsistency in the strength of the hits delivered to the mannequin. Our study seeks to simulate a live environment, which would not have a standardized force due to human inconsistency. A previous concussion study measuring impact forces sustained during live football practices had similar inconsistencies in the strength of each hit but was able to overcome this by collecting large amounts of data points [27]. Similarly, we addressed this hurdle in our study by gathering more data points to yield a meaningful 95% CI. However, further increasing the number of hits may have the potential to narrow the CIs even more. Another limitation may be from our experimental setup as we focused only on measuring direct vertical strikes to the kendo helmets. The sensor device within the mannequin could not measure other rotational or horizontal forces from hits to the side of the head and thrusts to the throat, which are considered other valid strikes in kendo. Furthermore, another factor we did not consider was the helmet fit since wearing oversized helmets has been proven to be less protective than well-fitting helmets [24]. While the helmets we tested for our study were the same size, future studies could look into testing how well a kendo helmet fits and can affect the strength of impact forces on the helmet. Finally, the number of participants in this study was relatively small. Although the data analysis yielded statistically significant results, the results may still be subject to a potential sampling size bias.

Chronic traumatic encephalopathy (CTE), caused by repeated head impact with or without concussion, is well reported in the literature [28,29]. As shown in our study, straight downwards strikes to a protected head in kendo are well protected from concussion, but the long-term effects of sub-concussions, head impacts that cause non-immediate symptoms, were not addressed in this study. In comparison with our study, which had an average linear acceleration of 13.3 g-forces, one study showed that repeated head linear accelerations of 33.2 g-forces by soccer balls are at risk of enough to significantly reduce King-Devick Test (KDT) performance, an exam that requires memory, concentration, anticipation, and language [28]. It is unclear if kendo practitioners will experience a similar decrease in KDT testing performance with repeat shinai strikes.

Future research could address the potential risk of brain injury resulting from chronic sub-concussive blows in kendo, measuring the protection provided by pads employed inside various helmet sizes, and exploring a wider range of materials used in kendo paddings, such as air, and polyurethane foam.

A reviewer from our prior study expressed curiosity about potential strength differences in the striking forces between American and Japanese kenshi. Anecdotally, it has been suggested that American practitioners and beginners tend to deliver harder strikes with less tenouchi, a kendo technique that involves gripping the shinai handle to control and reduce the force upon impact. With this possibility in mind, for future research, our team was interested in reproducing this experiment with a larger database. Furthermore, we could take the experiment one step further by incorporating a panel of at least three shinpan (judges) to assess whether a strike is valid based on sound and technique. Then compare the striking forces between the ones that score points and the ones that do not score points.

## Conclusions

The results indicate that various pads and bogu stitching patterns used in kendo sufficiently protect against concussions. There are, however, significant differences in performance when viewed comparatively. Investing in a PP would provide more protection than any traditional thick, small, or wide cotton pad. Furthermore, the higher-end helmets, which generally have smaller stitching patterns, provided significantly less protection than lower-end helmets. With that said, helmet stitching patterns between 2 mm and 9 mm are sufficient to protect against concussions. Therefore, purchasing a higher-end helmet does not necessarily provide more protection, and may even provide less.

Our study demonstrates that male kendo practitioners strike with greater force (7.9%) compared to female practitioners. However, with proper protection, strikes received by male or female practitioners remained well below the concussion threshold. The same can be stated for veteran dan-level practitioners, as they tended to hit with higher force (8.8%) when compared to beginner Kyu-level practitioners in this study.
